# Disruption of *FGF5* in Cashmere Goats Using CRISPR/Cas9 Results in More Secondary Hair Follicles and Longer Fibers

**DOI:** 10.1371/journal.pone.0164640

**Published:** 2016-10-18

**Authors:** Xiaolong Wang, Bei Cai, Jiankui Zhou, Haijing Zhu, Yiyuan Niu, Baohua Ma, Honghao Yu, Anmin Lei, Hailong Yan, Qiaoyan Shen, Lei Shi, Xiaoe Zhao, Jinlian Hua, Xingxu Huang, Lei Qu, Yulin Chen

**Affiliations:** 1 College of Animal Science and Technology, Northwest A&F University, Yangling 712100, China; 2 MOE Key Laboratory of Model Animal for Disease Study, Model Animal Research Center of Nanjing University, National Resource Center for Mutant Mice, Nanjing 210061, China; 3 School of Life Science and Technology, ShanghaiTech University, Shanghai 201210, China; 4 Shaanxi Provincial Engineering and Technology Research Center of Cashmere Goats, Yulin 719000, China; 5 Life Science Research Center, Yulin University, Yulin 719000, China; 6 College of Veterinary Medicine, Northwest A&F University, Yangling 712100, China; Utah State University, UNITED STATES

## Abstract

Precision genetic engineering accelerates the genetic improvement of livestock for agriculture and biomedicine. We have recently reported our success in producing gene-modified goats using the CRISPR/Cas9 system through microinjection of Cas9 mRNA and sgRNAs targeting the *MSTN* and *FGF5* genes in goat embryos. By investigating the influence of gene modification on the phenotypes of Cas9-mediated goats, we herein demonstrate that the utility of this approach involving the disruption of *FGF5* results in increased number of second hair follicles and enhanced fiber length in Cas9-mediated goats, suggesting more cashmere will be produced. The effects of genome modifications were characterized using H&E and immunohistochemistry staining, quantitative PCR, and western blotting techniques. These results indicated that the gene modifications induced by the disruption of *FGF5* had occurred at the morphological and genetic levels. We further show that the knockout alleles were likely capable of germline transmission, which is essential for goat population expansion. These results provide sufficient evidences of the merit of using the CRISPR/Cas9 approach for the generation of gene-modified goats displaying the corresponding mutant phenotypes.

## Introduction

Genome-editing technologies that enable efficient and precise genome manipulation in livestock species could facilitate in the improvement of productivity, disease resistance, and breeding capabilities, as well as biomedical studies. The usage of nuclease genome-editing technologies including zinc finger nucleases (ZFNs), transcription activator-like effector nucleases (TALENs), as well as the most recent techniques, clustered regularly interspaced short palindromic repeats (CRISPR)-associated system has facilitated in the generation of gene-modified livestock [1–5], as well as shed some light on the potential of precision editing of animal genomes. Yet, the phenotypic consequences of CRISPR/Cas9 mediated gene modifications in livestock have not been thoroughly explored.

We previously showed that the CRISPR/Cas9 system is feasible in generating genetically modified goats, 26 out of 98 (26.5%) founder animals were determined as Cas9-mediated goats [6]. In addition, the phenotypes induced by Cas9-mediated loss-of-function mutations were remarkably different, as well as the phenotypic traits transmitted to the next generations have not been established. Given the crucial role of fibroblast growth factor 5 (FGF5) in determining hair length in dogs [7, 8], cats [9], mice [10], and humans [11], we therefore investigated whether the disruption of *FGF5* in goats results in changes in hair length. In the present study, by investigating the effects of genetic modification using phenotypic and genotypic data, we demonstrated that the increased fiber length in cashmere goats was indeed caused by the knockout alleles in *FGF5*, and that these phenotypes have the potential to transmit to following generations.

## Results and Discussion

### Disruption of *FGF5* results in increased hair length in goats

**Fig 1:**
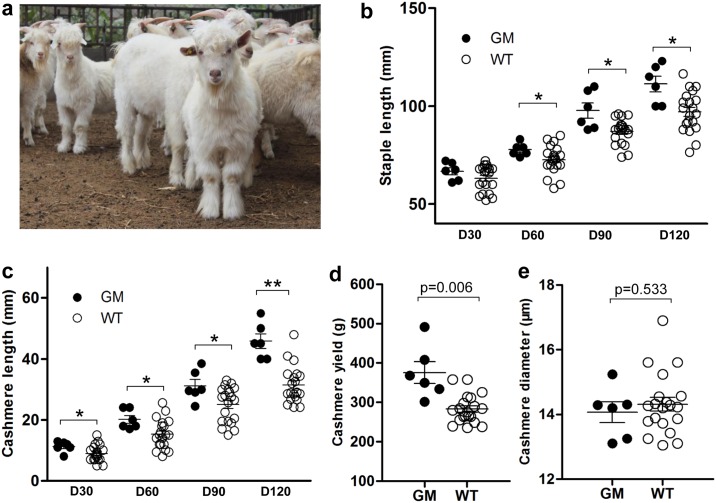
Evaluation of phenotypic changes in goats using CRISPR/Cas9 gene editing. **(a)** Cas9-mediated FGF5-disrupted goats at D160. (The Photo was taken by X. Wang). **(b)** Staple length between *FGF5*-disrupted (GM) and WT goats at different growth stages. **(c)** The length of cashmere between *FGF5*-disrupted (GM) and WT goats at different growth stages. **(d)** Differences in cashmere yield between *FGF5*-disrupted (GM) and WT goats at D120. **(e)** Differences in the diameter of cashmere fibers between *FGF5*-disrupted (GM) and WT goats at D120. * p < 0.05, ** p < 0.01, Student's *t*-test.

Cashmere goats are featured with a double coat consisting of the outer coarse hair produced by primary hair follicles (PHF) and the inner fine coat (cashmere) produced by secondary hair follicles (SHF) [12]. Because the *FGF5* gene is the master regulator controlling hair length, we therefore measured the length of both coat hair and inner hair of animals we obtained from a previous study [6], starting from day 30 (D30) after birth and at every 30 days throughout the trial. The gene-modified animals grew apparently normally and remain in relatively good health (Fig 1a). Only 6 out 19 live animals were regarded as single gene disruption (Table 1), we therefore analyzed the hair phenotypes of these six animals with controls. The results showed that, except the staple length (length of coat hair) at D30, the length of the coat hair and cashmere in goats with only *FGF5* disruption (n = 6) was significantly longer than that in the control group (n = 20) from D30 to D120 (p < 0.05) (Fig 1b and 1c). Besides the longer cashmere length, cashmere yield of Cas9-mediated animals was higher than that of the WT at D120 (p = 0.018), with an average increase of 92.75 g cashmere for each animal (Fig 1d), indicating that the Cas9-mediated animals were more productive in cashmere yield. We also compared the diameters of the cashmere fibers of four-month-old gene-modified animals and that of the four-month-old control animals, which showed no significant differences (p = 0.533) (Fig 1e). Evaluation of cashmere yield, diameter, and length of cashmere fibers, which are the three most economically important traits in fiber-producing goats, has shown that Cas9-mediated goats targeting the *FGF5* gene exhibited increased fiber length and cashmere yield, whereas cashmere diameter did not change.

**Table 1:** Summary of the 19 alive gene-modified animals.

No.	Animal ID	Targeting information^a^			No. of clones	Mutations in *FGF5* loci^b^	mono or biallelic
		*FGF5*	*MSTN*_sg1	*MSTN*_sg2			
1	#9				8	(-27, -5)	biallelic
2	#19				6	(+1M2, -21), -41M6	biallelic
3	#21				10	(-26, -57,+10), (-218,+28), WT	monoallelic
4	#23				7	-9, (-169M5), -190, WT	monoallelic
5	#40				9	(-8, -75), -175	biallelic
6	#41				6	(-8, -75), -175	biallelic
7	#42				-	-	-
8	#43				9	(-1M1, -4), (-8, -27), (-8, -57,+10), (-176, +7)	biallelic
9	#46				10	(-26, -57+10), (-176,+7), -190, (-218, +28), WT	monoallelic
10	#47				9	(-176, +7), (-218, +30), WT	monoallelic
11	#53				9	-43, WT	monoallelic
12	#70				9	-9, (-26, -57+10), (-183, +6), (-218, +28), WT	monoallelic
13	#73				9	+2, -1M2, WT	monoallelic
14	#76				8	-5, -11, WT	monoallelic
15	#81				8	+1, -169	
16	#82				7	-1, (-3, -1), WT	monoallelic
17	#84				5	(-42M3, -33), -189	
18	#93				13	(+2, -9M1), (-10, -9), (-26, -57, +10), (-176, +7), (-220, +30), (-218, +28)	biallelic
19	#99				7	(+2, -9M1), -169, (-191+24), WT	monoallelic

^a^The shadows indicate the occurrence of disruption at an given locus.

^b^‘-n’ indicates n bp deletion, ‘+n’ indicates n bp insertion, ‘Mn’ represents the number of replaced mutations, WT represent wild-types.

Although the average cashmere length of the *FGF5*-disrupted groups is significantly longer than that in the WT animals, varied fiber length from founders were observed, and the cashmere length from some of the WT individuals is even longer than founder animals. A wide range of somatic mosaicism and allele complexity in founders can be explained by mosaic animals resulting from RNA injections into one-cell stage zygotes during CRISPR/Cas9-mediated mutagenesis [13–15], we therefore expect further studies to breed these founders for producing homozygous offspring.

### Morphological characterization of goat skins modified by CRISPR/Cas9

**Fig 2:**
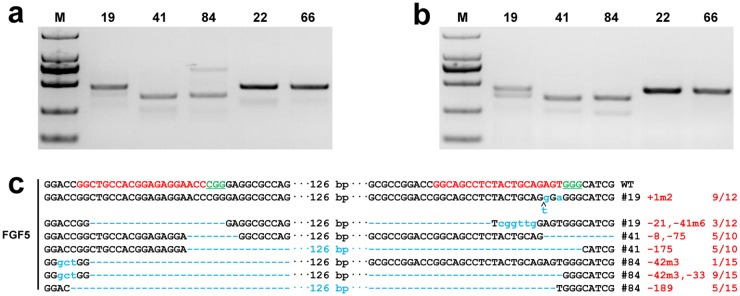
Genotypes of Cas9-mediated *FGF5* modified and WT goats. **(a)** PCR products of the targeted region of *FGF5* from three founder goats (#19, #41, #84) and two WT goats (#22, #66) at 120 days old. **(b)** Detection of sgRNA:Cas9-mediated on-target cleavage of *FGF5* by using the T7E1 cleavage assay. All PCR products from **(a)** were subjected to the T7E1 cleavage assay. **(c)** Sequencing results of modified *FGF5* loci detected in goat skins of founders (#19, #41, #84), 9/12 represents 9 out of 12 clones showing the given genotype.

We previously investigated the genome targeting in the tissues representing three germ layers of goat fetuses [6], which has provided evidence that mutations extensively occurred across various tissues. To verify whether the phenotypes were induced by Cas9-mediated mutations, we collected skin tissues from three 120-day-old goats (#19, #41, and #84), which were investigated by T7E1 cleavage and Sanger sequencing. The T7E1 assay showed cleavage bands of *FGF5* in the skins from different animals (Fig 2a & 2b). Sequencing further confirmed that the skin tissues were mutated at the cleaved site of variable mutation sizes for each animal at different clones (Fig 2c), thereby confirming that nearly the same mutations occurred in somatic tissues.

**Fig 3:**
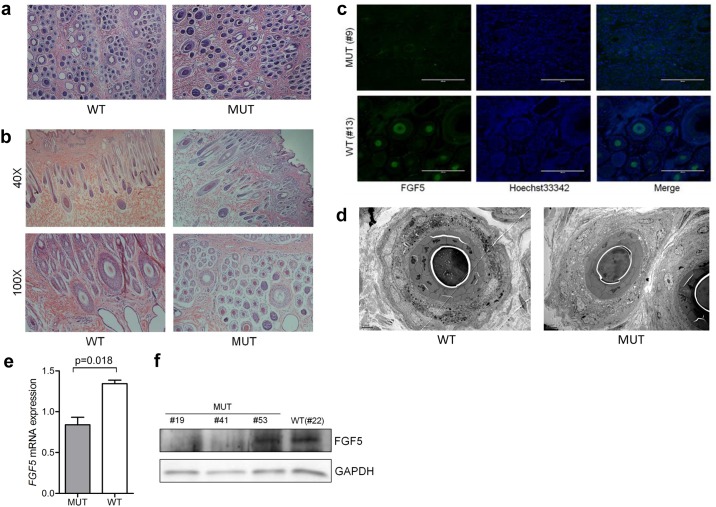
Morphological analyses of skin tissues from Cas9-mediated *FGF5* disrupted and WT goats. **(a)** H&E staining shows HF morphology in the skin of an aborted *FGF5* gene-modified (MUT) goat and an aborted WT goat. Scale bar = 200 μm. **(b)** H&E staining shows HF morphology in the skin of *FGF5* gene-modified (MUT) (#9) and WT goats at D120. **(c)** Immunohistochemistry of skin tissues from MUT and WT goats. Scale bar = 200 μm. **(d)** TEM analyses of HF from the skin of at 120-days old goats. Scale bars: left = 5 μm. **(e)** Quantitative RT-PCR analysis of *FGF5* in the skin of Cas9-mediated (MUT) and WT goats. Data are expressed as the mean ± SD. **(f)** Western blot analysis using anti-FGF5 and anti-GAPDH (loading control) antibodies.

Subsequently, to analyze the effect of CRISPR/Cas9-mediated genomic editing on the development of goat skin, we performed histological analysis using skin tissues from both aborted and live *FGF5*-mutated goats and the corresponding WT (aborted and live animals). Hematoxylin and eosin (H&E) staining demonstrated that there were more secondary hair follicles (SHF) in the skin of *FGF5*-disrupted goats (both in aborted and 120-day-old goats) than that in the WT (Fig 3a & 3b). The ratio of SHF/PHF in *FGF5*-disrupted goats was significantly higher than that in the WT (p < 0.01) (Figure A in S1 File), meaning more cashmere will be generated in *FGF5*-disrupted goats. In addition, the diameter of both primary hair follicles (PHF) and SHF from *FGF5*-mutated goats (both in aborted and 120-day-old goats) were larger than that in the WT (Fig 3a & 3b). Similar to other mammals, the PHF in cashmere goat skins produced guard hair, and the SHF produced fine fibers (cashmere) in a seasonal and cyclical manner [16, 17]. A previous report showed that the increased size of HF orifices is essential to providing more nutrition to HF during the anagen phase of rapid cell division [18], thereby resulting in longer fibers in *FGF5*-disrupted goats.

To further verify whether knocking out the *FGF5* gene would influence the expression level and the location of FGF5 expression at the tissue level, we performed immunofluorescence staining using the antibody against the FGF5 protein in *FGF5*-edited and WT goats. The results showed that the expression of *FGF5* was significantly downregulated in gene-modified goats compared to that of the WT goats, and the location of the FGF5 protein was not altered between the gene-modified and WT goats (Fig 3c). Transmission electron microscopy (TEM) was used to investigate the subtle changes in the SHF of goat skin at high resolution. TEM analyses confirmed that the structure of SHF in *FGF5*-disrupted goats were the same at that in WT (Fig 3d), suggesting that genetic modification did not affect the integrity of SHF, and therefore may not influence SHF cycling and hair differentiation in cashmere goats. Furthermore, no apparent morphological changes were observed in the muscles beneath the skin tissues between *FGF5*-disrupted (#19) and WT (#28) goats through H&E staining (Figure B in S1 File) and TEM (Figure C in S1 File).

In sum, these morphological findings suggest that Cas9-mediated goats targeting the *FGF5* gene results in an increase in the number of SHF in goat skin, whereas no changes in the integrity of SHF were observed. Further histological characterization confirmed no aberrant change was observed in muscle under the tested skins.

### Molecular characterization of goat skins modified by CRISPR/Cas9

We next sought to validate whether the expression of the *FGF5* gene was inhibited or downregulated at the transcriptional level. Specific primers encompassing the target sites of sgRNAs were designed, and quantitative RT-PCR was conducted using cDNA isolated from the skin of both gene-modified (n = 3) and WT (n = 3). Compared to the WT, the expression of *FGF5* in gene-disrupted goats was markedly reduced (p = 0.018) (Fig 3e), which suggested that the skin of gene-modified animals had limited FGF5 transcription. This inverse correlation was in agreement with a recent finding [19], and confirms the relevance of this regulatory mechanism to the development of goat skin. Western blotting was subsequently implemented to confirm the lack of protein expression in three randomly sampled gene-disrupted animals (#19, #41 and #53). Western blotting using skin tissues from *FGF5-*disrupted goats, as expected, showed almost no expression of FGF5 at the protein level in the tissues of #19 and #41, but not in #53 (Fig 3f), presumably because #19 and #41 are homozygous (biallelic) while #53 is heterozygous (monoallelic) (Table 1). Quantitative PCR and western blotting analyses indicated that *FGF5* was expressed at low levels in both transcriptional and protein levels in Cas9-mediated gene-modified goats, further indicating the successful disruption of the *FGF5* gene. Given the fact that the phenotypes and genotypes of gene-modified goats remarkably changed and that *FGF5* was differentially expressed at the transcriptional and protein levels, we therefore concluded that the observed phenotypes were most possibly caused by the designed disruption of *FGF5*.

### Germline transmission of mutant alleles

**Fig 4:**
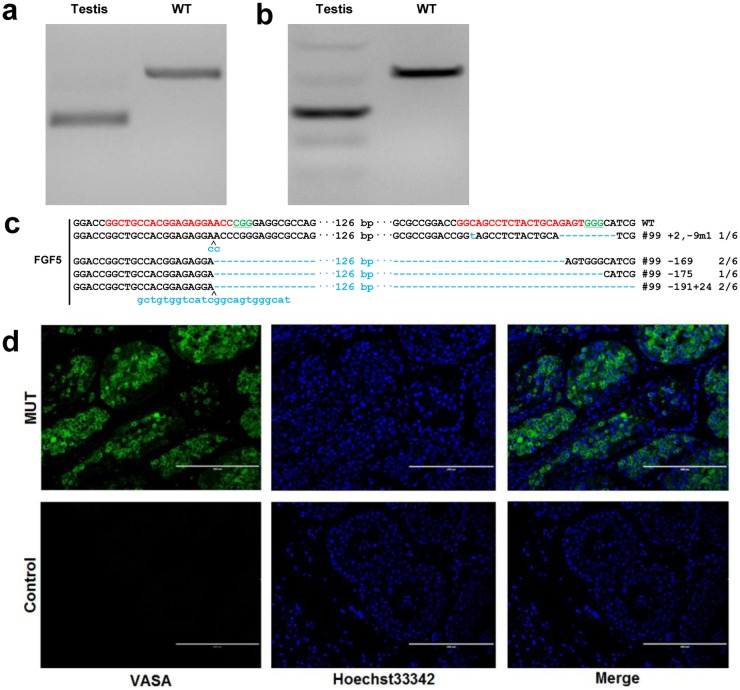
Detection of germline transmission in the testis of *FGF5*-disrupted goats. **(a)** PCR products of the targeted region of *FGF5* in testis from founder goat #99; **(b)** Detection of sgRNA:Cas9-mediated on-target cleavage of *FGF5* by T7E1 cleavage assay. PCR products from (a) were subjected to T7E1 cleavage assay. **(c)** Sequencing results of modified *FGF5* loci detected in testis. **(d)** Immunostaining analysis of biopsied testis of the founder (#99) at 120-day-old, confirmed by germ cell specific marker VASA. Germ cells from gonads of founders were stained with an anti-VASA antibody (green) and Hoechst 33342 (blue). VASA positive cells are germ cells. VASA negative cells were set aside as the negative control. Scale bar = 200 μm.

Efficient transmission of mutations introduced by sgRNA:Cas9 to the next generations is critical for population expansion of gene-modified goats and other breeding purposes. We have previously shown that the cleavage of the *MSTN* and *FGF5* genes did occur in the testis of founder animals through PCR and the T7E1 cleavage assay [6], indicating that Cas9-mediated targeting was integrated into the testis of founder animals. We here extracted DNA from biopsied testis of founder #99 and subject to T7E1 cleavage assay and sequencing (Fig 4a & 4b), Sanger sequencing confirmed PCR and T7E1 cleavage assay (Fig 4c). Cas9-mediated modification in the testis resulted in multiple types of variations, implying that Cas9-mediated genome targeting was successfully integrated into the testis, which in turn endows the germ cells the capacity to transmit the superior traits to the next generation. To further examine whether Cas9-mediated modifications occurred in the germ cells, testis from founder #99 were used for immunostaining analysis. The existence of germ cells in the testis was supported by immunostaining with a germ cell specific marker, VASA [20] (Fig 4d).

**Fig 5:**
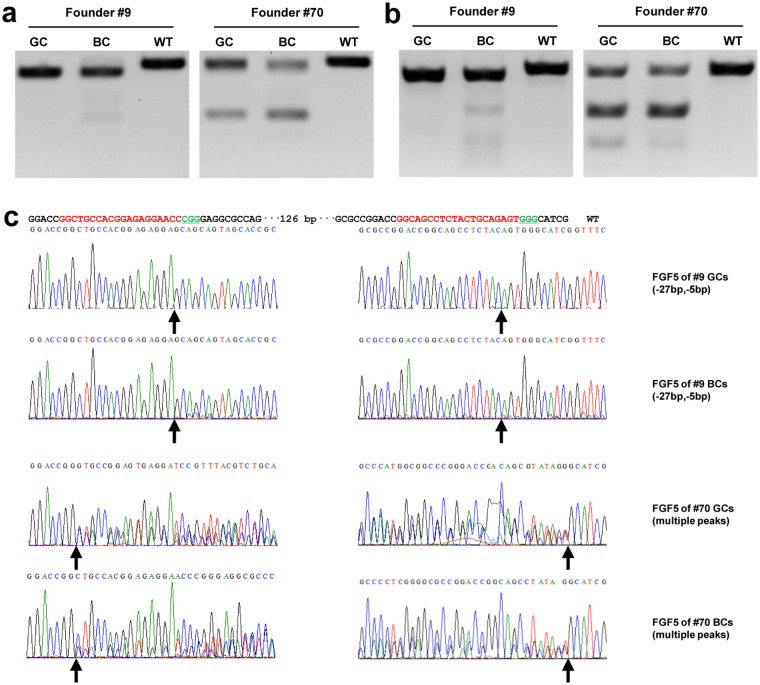
Germline transmission detection in the germ cells of *FGF5*-disrupted goats. **(a)** PCR products of the targeted region of *FGF5* in germ cells (GCs) and blood cells (BCs) from founder goats (#9 and #70) at 120 days old. **(b)** Detection of sgRNA:Cas9-mediated on-target cleavage of *FGF5* by T7E1 cleavage assay. All PCR products from (a) were subjected to T7E1 cleavage assay. **(c)** Sequencing results of modified *FGF5* loci detected in goat germ cells (GCs) and blood cells (BCs).

In addition, we extracted the germ cells from the testis of two founders (#9 and #70) and subject to T7E1 cleavage and PCR product sequencing. The T7E1 cleavage bands of the *FGF5* gene were observed in germ cells from goat testis (Fig 5a & 5b). The sequencing results confirmed that the germ cells from testis had the same mutations as somatic tissues, such as 27 bp and 5 bp deletions were found in founder #9 in both germ cells and blood cells (Fig 5c). The existence of germ cells with target mutations in testis further demonstrated the Cas9-mediated target gene mutations are most possibly heritable to the following generations. Given it takes at least around 12 months for cashmere goats to reach sexual maturity and followed with a gestation period, we are unable to perform breeding at the moment, we therefore expect future studies to investigate the germline transmission in the F2 generations.

In summary, our results show that the CRISPR/Cas9 system increases the number of SHF and cashmere length, but not fiber diameter in goats. Germline transmission analysis further demonstrated that likely the CRISPR/Cas9-mediated genomic modifications can be transmitted through goat germlines to its offspring. Together with the findings of our previous studies, we provide evidence that highlights the merits of sgRNA:Cas9-mediated genome editing as a powerful approach in accelerating the genetic improvement of goats.

## Materials and Methods

### Ethnic statement

The production of genetically modified goats via the CRISPR/Cas9 approach was described by Wang *et al* [6]. The gene-modified and WT goats were housed in the Shaanbei Cashmere Goat Farm of Yulin University under the same maintenance conditions, specially, natural light cycles and regular feeding schedules were used. All protocols involving the use of animals were in accordance with approved guidelines of the Animal Care and Use Committee of the Northwest A&F University (Approval ID: 2014ZX08008-002). To obtain biopsied tissues from goats, 2–3 mL procaine (1%) was injected into skin/muscle tissues for local anesthesia. All efforts were made to minimize animal suffering and to reduce the number of animals used.

### Phenotypic data

Animals were raised under the same conditions and fed the same types of feed throughout the trial. The feed were adjusted with respect to growth stages after weaning at D60. The staple length and cashmere length were sampled every 30 days starting at D30 after birth, and were measured using conventional method. The cashmere yield and diameter of cashmere were measured using samples obtained at D120. Pairwise data were analyzed using the student’s *t*-test.

### T7E1 assay and PCR sequencing

The T7E1 cleavage assay was conducted as described by Shen et al [21]. Briefly, targeted fragments were amplified using PrimerSTAR HS DNA polymerase (TaKaRa) from the isolated genomic DNA, and then purified with a PCR Cleanup kit (OMEGA). The purified PCR products were denatured and re-annealed using NEBuffer 2 (NEB) via a BioRad thermocycler. The PCR products were digested with T7E1 (NEB, Beijing, China) for 30 min at 37°C, and were separated on a 2.5% agarose gel. PCR products with mutations detected by the T7E1 assay were sub-cloned into a T vector (TaKaRa). Total 15 colonies for every sample were randomly picked, and then sequenced using the M13-47 primer (5'-CGC CAG GGT TTT CCC AGT CAC GAC-3').

### H&E and immunofluorescence staining

A portion of the skin tissues, as well as the subcutaneous muscle tissues from backside of the Cas9-mediated gene modified and WT goats were biopsied. Tissue biopsies were immediately fixed in 4% paraformaldehyde at 4°C overnight, and then were embedded in paraffin using standard laboratory procedures. After cutting the samples into 2 μm slices, the staining was carried out with hematoxylin-eosin. In addition, the SHF/PHF number ratio was determined in six non-overlapping fields using a light microscope and a 4× objective. Three *FGF5*-disrupted goats (#9, #19, and #23) and three WT (#4, #13, and #18) were used for SHF/PHF ratio measurement. The tissue sections were de-waxed, rehydrated, and stained using standard immunohistochemical protocols. For immunofluorescence, the anti-FGF5 (Sigma-Aldrich, 1:300) primary antibody was used. The sections were then counterstained with Hoechst 33342 and analyzed by confocal laser microscopy.

### TEM analyses

Skin and muscle tissues from both *FGF5*-disrupted and WT goats were biopsied. For TEM analysis, samples were prepared as previously described [22]. Briefly, the skin and muscle sections were post-fixed with 1% (w/v) glutaraldehyde in 0.1 M phosphate buffer (PB) (pH 7.4) for 10 min and washed in distilled water. After silver enhancement by using an HQ Silver Kit (Nanoprobes), the sections were incubated at room temperature with a 1:50-diluted Elite ABC Kit (Vector, Burlingame, CA) in 0.05 M TBS for 6 h and then further incubated at room temperature with 0.05 M Tris-HCl (pH 7.6) containing 0.02% (w/v) 3,30-diaminobenzidine tetrahydrochloride (DAB, Tokyo, Japan) and 0.003% (v/v) H_2_O_2_ for 20–30 min. Subsequently, the sections were placed in 0.1 M PB (pH 7.4) containing 1% (w/v) O_s_O_4_ for 1 h and then counterstained with 1% (w/v) uranyl acetate in 70% ethanol for 1 h. After dehydration, the sections were mounted on silicon-coated glass slides and flat embedded in epoxy resin (Durcupan, Buchs, Switzerland). Upon resin polymerization, small pieces of tissues were cut out from the flat-embedded sections, and selected tissues were cut into 60-nm-thick sections using an ultramicrotome (Reichert-Nissei Ultracut S, Vienna, Austria). Sections were examined under a JEM-1230 electron microscope (JEOL, Tokyo, Japan) and captured by using a Gatan 832 (Gantan, Pleasanton, CA).

### Quantitative RT–PCR analysis

Total RNA was extracted from skin tissues of three Cas9-mediated and three WT goats using TRIzol (TaKaRa) and treated with RNase-free DNase I (TaKaRa) following the manufacturer's protocol. The resulting RNA was reverse transcribed using an M-MLV reverse transcriptase kit (TaKaRa). Each qRT-PCR assay was replicated at four times with three independent RNA preparations, and the expression for each sample was normalized to the endogenous control gene, *GAPDH*. Relevant PCR primer sequences, including gene-specific primers across the knockout regions of the *FGF5* gene, are given in Table A in S1 File. Twenty-microliter reactions were performed in triplicate by using a BioRad thermocycler, with at least three biological replicates, to determine each data point. The relative gene expression levels were calculated using the 2^−ΔΔCt^ method. The presence of significant differences in expression was calculated with student’s t-test.

### Western blot analysis

Skin tissues were subjected to total protein extraction using a ProteoJET Membrane Protein Extraction Kit (Fermentas), and then quantified using the Bradford assay. Equal amounts of soluble protein were separated by SDS/PAGE and transferred onto a polyvinylidene difluoride membrane (PVDF, Roche). Immunoblotting was conducted using antibodies specific for FGF5 (1:1000, Sigma-Aldrich) and anti-GAPDH (1:1000, Sigma-Aldrich). Primary antibodies were visualized using a fluorescence imager system (Sagecreation). Variations in sample loading were corrected by normalizing.

### Germline transmission detection

Germ cells from the testis of two founders were obtained by using biopsy forceps (Olympus FB-11K). Spermatogonia were then extracted and cultured. The PCR products were subject to T7E1 cleavage assay and followed with Sanger sequencing to confirm.

The existence of germ cells from biopsied testis of gene-modified animals (120 days old) was examined by immunostaining with VASA, which is a germ cell specific marker. Immunofluenscence staining was conducted as previous described [23]. In brief, rat anti-Daphnia VASA antibody were raised against bacterially expressed N-terminal region of the VASA protein (residues 1–332) and affinity purified for immunostaining. In the experiment, a dilution of 1:500 of the primary antibody (1:500, Abcam) was used for most of the reactions.

## Supporting Information

S1 File Figure A. The ratio number of SHF/PHF in FGF5-disrupted goats (#9, #19, and #23) was significantly higher than that in the WT (#4, #13, and #18). Figure B. H&E staining of muscle tissues from the mutant (#19) and wildtype (#28) goats. Figure C. TEM analysis of muscles from the mutant (#19) and wildtype (#28) goats. Table A. Primers used for qRT-PCR.
(DOC)
