# Correction: Disruption of *FGF5* in Cashmere Goats Using CRISPR/Cas9 Results in More Secondary Hair Follicles and Longer Fibers

**DOI:** 10.1371/journal.pone.0167322

**Published:** 2016-11-22

**Authors:** Xiaolong Wang, Bei Cai, Jiankui Zhou, Haijing Zhu, Yiyuan Niu, Baohua Ma, Honghao Yu, Anmin Lei, Hailong Yan, Qiaoyan Shen, Lei Shi, Xiaoe Zhao, Jinlian Hua, Xingxu Huang, Lei Qu, Yulin Chen

The title of the first subsection in the Materials and Methods is incorrect. The correct title is: Ethics Statement.
