# Supplementary material for: Disruption of FGF5 in Cashmere Goats Using CRISPR/Cas9 Results in More Secondary Hair Follicles and Longer Fibers
Source: PLoS One. 2016 Oct 18;11(10):e0164640. doi: 10.1371/journal.pone.0164640 (PMC5068700; doi:10.1371/journal.pone.0164640)
Supplement: S1 File — Figure A. The ratio number of SHF/PHF in FGF5-disrupted goats (#9, #19, and #23) was significantly higher than that in the WT (#4, #13, and #18). Figure B. H&E staining of muscle tissues from the mutant (#19) and wildtype (#28) goats. Figure C. TEM analysis of muscles from the mutant (#19) and wildtype (#28) goats. Table A. Primers used for qRT-PCR. (DOC) [file pone.0164640.s001.doc]

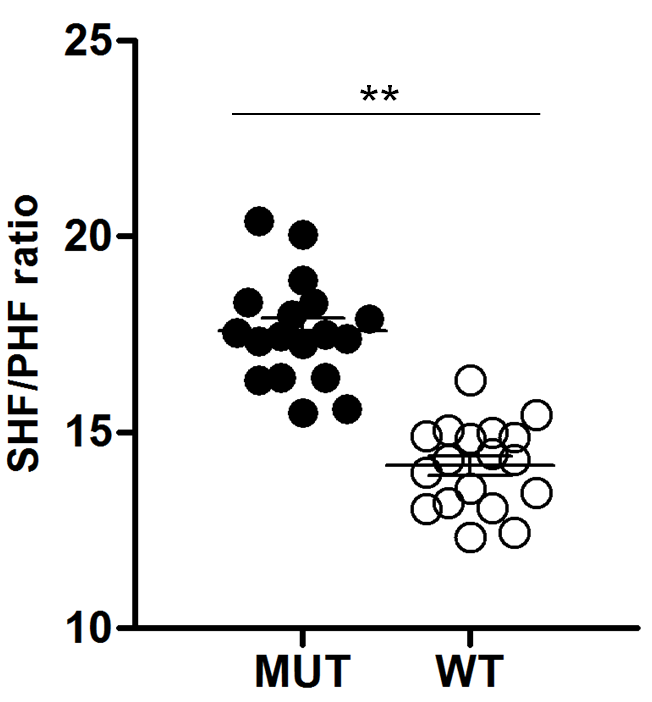


**Figure A.** The ratio number of SHF/PHF in *FGF5*-disrupted goats (#9, #19, and #23) was significantly higher than that in the WT (#4, #13, and #18) (**, p < 0.01, student’s t-test).


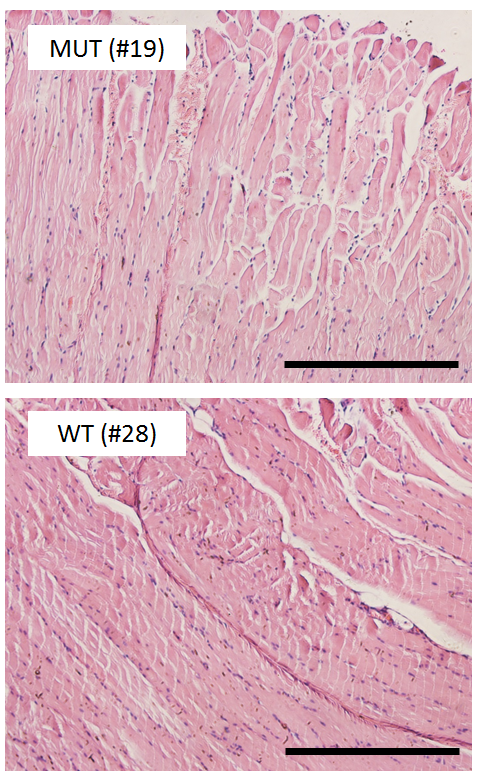


**Figure B.** H&E staining of muscle tissues from the mutant (#19) and wildtype (#28) goats. Scale bars=200 μm.


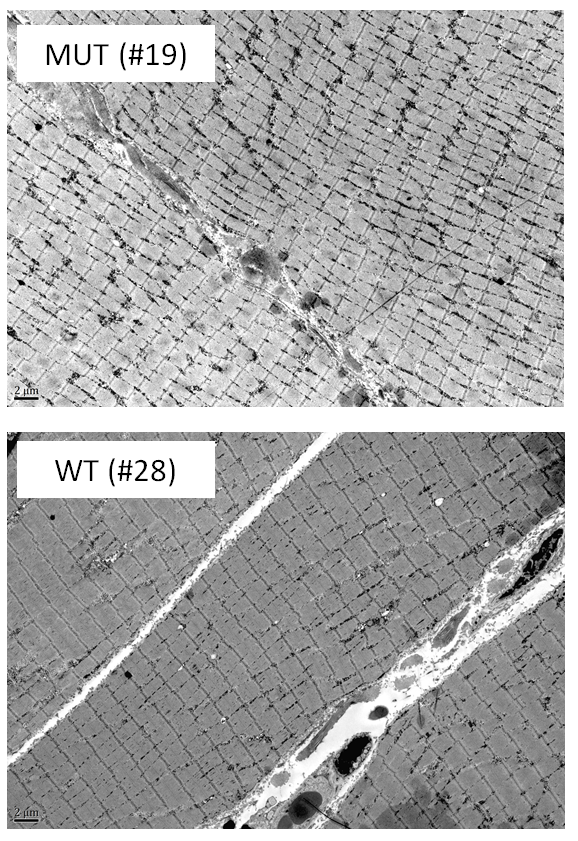


**Figure C.** TEM analysis of muscles from the mutant (#19) and wildtype (#28) goats. Scale bars=2 μm.

**Table A.** Primers used for qRT-PCR.

| Gene | Sequence | Product size (bp) |
| --- | --- | --- |
| *FGF5* | F: ACCGCGTCTTCCTCTTCTTC | 256 |
|  | R: TTGTTGCTGAAAACTCCTCGT |  |
| *GAPDH* | F: TCCGTTGTGGATCTGACCTG | 157 |
|  | R: AAGAGTGAGTGTCGCTGTTGAAG |  |
